# Bacteremia in febrile cancer patients in Uganda

**DOI:** 10.1186/s13104-019-4520-9

**Published:** 2019-07-30

**Authors:** Margaret Lubwama, Warren Phipps, Christine F. Najjuka, Henry Kajumbula, Henry Ddungu, Joyce B. Kambugu, Freddie Bwanga

**Affiliations:** 1 Department of Medical Microbiology, Makerere University, Kampala, Uganda; 2 Vaccine and Infectious Disease Division, Fred Hutchinson Cancer Research Center, Seattle, WA USA; 3 Department of Medicine, University of Washington, Seattle, WA USA; 4 Uganda Cancer Institute, Kampala, Uganda; 5 Department of Immunology and Molecular Biology, Makerere University, Kampala, Uganda

**Keywords:** Bacteremia, Neutropenia, Antimicrobial resistance, Antibiotics, Cancer, Uganda, Sub-Saharan Africa

## Abstract

**Objective:**

The aim of this study was to determine the predominant bacterial species causing bacteremia among febrile cancer patients, and their antibacterial resistance profiles at the Uganda Cancer Institute.

**Results:**

We enrolled in-patients with a documented fever (≥ 37.5 °C). Bacteria from positive blood cultures were identified using standard methods biochemically. Antibacterial susceptibility testing was performed with the Kirby–Bauer disc diffusion method. From a total of 170 febrile episodes, positive blood cultures were obtained from 24 (14.1%). A positive culture was more likely to be obtained from a patient with neutropenia (P = 0.017). Of 22 (66.7%) Gram-negative bacteria isolated, half were *E. coli* (n = 11). Gram-negative compared to Gram-positive bacteria were most likely to be isolated from patients with a hematologic malignancy (P = 0.02) or patients with neutropenia (P = 0.006). Of the isolated Enterobacteriaceae 85% (n = 20) were resistant to three or more classes of antibiotic and 41% (n = 7) had extended spectrum beta-lactamases. Of the 11 Gram-positive bacteria isolated, the *S. aureus* isolate was methicillin resistant but susceptible to vancomycin. Multidrug resistant Gram-negative bacteria are the main cause of bacteremia in febrile cancer patients at the Uganda Cancer Institute. There is need for ongoing microbial surveillance, infection prevention and control, and antibiotic stewardship programs.

## Introduction

Patients with cancer, a growing problem in sub-Saharan Africa (SSA) are at increased risk for infections for several reasons such as immunodeficiencies related to several cancers, immunosuppressive anticancer chemotherapy and frequent interaction with health care settings for treatment [1]. Neutropenia is a particularly important risk factor associated with bloodstream infections in up to 25% of cancer patients [2], with death rates as high as 24% in high-income countries and 33% in low-middle-income countries (LMIC) [3–5]. Timely and appropriate empiric treatment of suspected infection, particularly among cancer patients with febrile neutropenia, is thus a cornerstone of oncology management.

Current recommendations of empirical antimicrobial management in cancer patients in the US and Europe are based in part on surveillance of bacterial isolates, which importantly have changed over the last 40 years, with a shift from predominantly Gram-negative etiology to Gram-positive etiology [6, 7]. In the less developed countries such as India, the predominant etiology over the years has been Gram negative bacteria. However, there has been a noted change in the trend in antimicrobial susceptibility patterns over the years with an increase in antimicrobial resistant bacteria. This has required a switch in empirical antibiotics used [8]. In SSA settings, baseline data on bacterial species involved in cancer-associated bacteremia and resistance remain very limited. Few studies have evaluated the etiology of bacteremia among cancer patients in Africa [9–12]. The microbiologic spectrum of bacteremia in SSA likely differs from that of the US or Europe due to differences in medical practice patterns, high endemic infection burden in the population, limited regulation of antibiotic use, and co-morbid conditions such as HIV and malnutrition [9].

Because an understanding of local bacterial isolates and resistance patterns is essential for guiding effective prophylaxis, empiric treatment and, ultimately, directed management, we undertook this study to identify the causes of bacteremia and to describe their susceptibility patterns among febrile cancer patients at the Uganda Cancer Institute (UCI), Kampala.

## Main text

### Methods

#### Study design, settings and population

We performed a cross-sectional study between March and November 2014 in which febrile episodes of cancer inpatients at the UCI were investigated for bacteremia. The UCI is the only national cancer referral center in Uganda, with an 80-bed inpatient facility. It is affiliated with the Makerere University College of Health Sciences and the Mulago hospital complex.

#### Participant recruitment and procedures

Eligible patients had a confirmed histological diagnosis of cancer and a temperature ≥ 37.5 °C. Neutropenia was defined as an absolute neutrophil count of ≤ 1000 cell/µL. We categorized patients as adults if ≥ 15 years of age. Patient characteristics were abstracted from the medical chart using a pre-prepared form. Patients were included more than once if they had another febrile episode after being afebrile for 72 h.

For each febrile episode, two blood samples were collected (one each from two peripheral sites) and placed in BACTEC aerobic bottles (Becton–Dickinson. USA). Adults had 5–10 mL of blood drawn while pediatric patients had 1–3 mL of blood collected.

#### Laboratory procedures

Samples were processed at the Makerere University Clinical Microbiology laboratory in the Department of Medical Microbiology. The blood culture bottles were placed into the BACTEC 9120 blood culture system (Becton–Dickinson, USA) for continuous monitoring according to manufacturer’s instructions. Samples from bottles that flagged positive were withdrawn for Gram stain and sub-cultured onto chocolate agar, blood agar and MacConkey agar plates. The plates were incubated for 18–24 h at 35–37 °C. Resulting colonies were subjected to further conventional biochemical tests for definitive identification. If organisms could not be identified using the conventional methods, the Phoenix Automated Machine (Becton–Dickinson, USA) was used, according to the manufacturer’s instruction.

#### Drug susceptibility testing

Antibiotic susceptibility testing was performed using the Kirby–Bauer disc diffusion method according to Clinical & Laboratory Standards Institute (CLSI) standards [13]. Screening for methicillin resistant *S. aureus* and coagulase negative staphylococci was carried out using the cefoxitin disc; inducible clindamycin resistance was detected using the D-zone test; and the double disc synergy test was used to detect extended spectrum beta-lactamase (ESBL) production by Gram-negative bacteria [14]. Coagulase negative staphylococcus, *Bacillus species* (spp.), and *Corynebacterium* spp. were considered “pathogens” if they grew in two of two collected blood culture bottles or if they grew in one bottle in a patient with clinical evidence of a bloodstream infection; otherwise they were considered contaminants. “Multidrug resistant organisms” were defined as organisms resistant to three or more classes of drugs [15]. Known control strains (*Escherichia coli* ATCC 25922, *Pseudomonas aeruginosa* ATCC 27853, *Klebsiella pneumonia* ATCC 700603 and *Staphylococcus aureus* ATCC 25923) were tested in parallel with the isolates from the specimen for quality control.

#### Statistical analysis

Categorical data were described as proportions. Analyses were carried out to compare the significance of difference in distribution by using Chi square test or Fisher’s exact test where appropriate. P-values of ≤ 0.05 were considered statistically significant. Statistical analysis was performed using Stata Version 14.0.

### Results

#### Participant characteristics

We observed 170 independent febrile episodes in 132 patients. Median participant age was 15 years (range 4 months to 70 years); 71 subjects (54%) were adults (Table 1). Eighty-two participants (62%) were male, and 87 (66%) had a hematological malignancy. Of 132 participants, 105 (80%) had a single febrile episode, 18 (14%) had two febrile episodes, 7 (5%) had three febrile episodes, and 2 (< 2%) had four febrile episodes. Of the 170 blood cultures obtained during the different febrile episodes, 24 (14.1%) were positive. One patient had a positive culture for different organisms from two separate episodes.

**Table 1:** Characteristics of patients

Characteristics	Patients (N = 132)		*P* value
	Positive culture (n = 23)	Negative culture (n = 109)	
Sex			
Male	14	68	0.89
Female	9	41	
Age			
Pediatric	10	51	0.77
Adult	13	58	
Cancer type			
Hematologic	17	70	0.37
Solid	6	39	
Neutropenic state (cells/µL)			
≤ 1000	16	46	0.017
> 1000	7	63	

#### Bacterial isolates

Nine positive cultures had a single Gram-negative bacterium, 8 had a single Gram-positive bacterium, and 7 were polymicrobial (Table 2). Of 33 bacteria isolated, 22 (66.7%) were Gram-negative and 11 (33.3%) were Gram-positive. Among the Gram-negative organisms, 20 (91%) were Enterobacteriaceae. Two rare pathogens isolated included *Rhodococcus equi* and *Cupriavidus pauculus*.

**Table 2:** Bacteria isolated from the 24 positive blood cultures

Organism isolated	Total
Gram negative organisms (n = 22)	
*E. coli*	11
*K. pneumoniae*	7
*Enterobacter* spp.	2
*Cupriavidus pauculus*	1
*Acinetobacter* spp.	1
Gram positive organisms (n = 11)	
*Bacillus* spp.	4
Coagulase negative	3
Staphylococcus	
* Corynebacterium* spp.	2
* S. aureus*	1
* Rhodococcus equi*	1
Total	33

#### Antimicrobial resistance

Among the Enterobacteriaceae, 17 (85%) were multidrug resistant of which 7 (41%) showed the ESBL phenotype (Table 3). Resistance to a carbapenem (imipenem) was observed in 4 (36.4%) *E. coli,* 4 (57.1%) *K. pneumoniae,* and 1 of the 2 *Enterobacter* spp. Non-Enterobacteriaceae, *Cupriavidus pauculus* and *Acinetobacter* spp., were multidrug resistant.

**Table 3:** Antibiotic resistance of enterobacteriaceae isolated

Class of drug	Drug	Organism number of resistant bacteria (%)		
		*E. coli* (N = 11) n (%)	*K. pneumonia*e (N = 7) n (%)	*Enterobacter* spp. (N = 2) n (%)
Beta-lactams	Ampicillin	10 (90.9)	7 (100)	2 (100)
	Augmentin	10 (90.9)	7 (100)	2 (100)
	Cefuroxime	8 (72.7)	7 (100)	2 (100)
	Ceftriaxone	7 (63.6)	7 (100)	2 (100)
	Ceftazidime	8 (72.7)	7 (100)	2 (100)
	Piperacillin/tazobactam	6 (54.5)	6 (85.7)	1 (50)
	Imipenem	4 (36.4)	4 (57.1)	1 (50)
Aminoglycoside	Gentamicin	8 (72.7)	5 (71.4)	2 (100)
Fluoroquinolone	Ciprofloxacin	9 (81.8)	6 (85.7)	2 (100)
Phenicol	Chloramphenicol	5 (45.5)	6 (85.7)	2 (100)
Sulfonamide	Cotrimoxazole	9 (81.8)	7 (100)	2 (100)

*Staphylococcus aureus* isolate was methicillin resistant (MRSA) and positive for inducible clindamycin resistance. The coagulase negative staphylococci were also methicillin resistant. All *Staphylococcus* species were sensitive to vancomycin. *Bacillus* spp. and *Rhodococcus equi* were susceptible to the antibiotics tested.

#### Risk factors for bacteremia

For analysis of clinical factors associated with bacteremia, we considered only the first positive blood culture observed from each participant. Out of the 23 single positive cultures included in analysis, 16 were from patients who had a neutrophil count of ≤ 1000 cell/µL and 7 were from patients who had a neutrophil count of ˃ 1000 cell/µL (*P* = 0.017). Seventeen positive cultures were from patients with hematologic malignancies and 6 were from patients with solid cancers (*P* = 0.37). Gram-negative bacteria were more likely to be isolated in patients with hematologic malignancies compared to solid tumors (P = 0.02), as well as in patients with a neutrophil count of ≤ 1000 cell/µL (P = 0.0006).

### Discussion

To our knowledge, this is first study of bacteremia among cancer patients in East Africa. The 14% rate of bacteremia observed at the UCI is comparable to 14.3% and 13.8% observed in pediatric oncology patients in South Africa in 2001 [10] and in 2012–2014 [12] respectively, but lower than the 22% rate reported in a study of cancer patients in Ghana [11]. Gram-negative bacteria were the most common organisms (66.7%). Studies from other LMIC also report Gram-negative bacteria to be the main etiology of bacteremia [16–18]. The relatively low proportion of Gram-positive bacteremia observed may be related to difference in practice patterns compared to high-income countries, including lack of indwelling catheters, which are a predisposing factor to Gram-positive bacteremia [19–21].

Importantly, our study shows that antimicrobial resistance is common among the bacteria isolated from patients at the UCI. Among the Enterobacteriaceae isolated, 85% were multidrug resistant, of which 41% showed the ESBL phenotype. Our finding of a high frequency of resistant organisms is similar to a study by Seni et al. which found that 81% of Enterobacteriaceae isolated from surgical infections at Mulago Hospital, which is in the same campus as the UCI, had the ESBL phenotype [22]. A high prevalence of ESBL-producing bacteria has also been reported among cancer patients in other LMIC, including a study carried out in India which detected ESBL in 84% of Gram negative bacteria isolated from acute leukemia and hematopoietic stem cell transplant patients [23]. ESBLs tend to be multidrug resistant, limiting the therapeutic options for infections caused by these organisms [24, 25]. Carbapenems are among the few treatment options for multidrug resistant Gram-negative bacteria. However, even though most of the ESBL organisms showed susceptibility to imipenem in our study, carbapenem resistance was observed in 4 (36%) *E. coli*, 4 (57%) *K. pneumoniae* and one (50%) of the *Enterobacter* spp. Similar results were observed in a Ugandan study of clinical samples obtained from Mulago hospital, which reported a carbapenemase prevalence of 22.4% of Enterobacteriaceae isolates, with the highest frequency (52.2%) among *Klebsiella pneumoniae* [26].

All the *Staphylococcus species* were methicillin resistant. MRSA has been reported in other studies that have been carried out in this region [27]. All the staphylococci isolated in this study were sensitive to vancomycin, making it the drug of choice for systemic infections where resistant staphylococcus species are suspected or confirmed.

We observed that specific patient sub-groups may be at higher risk of bacteremia. Neutropenia was highly associated with bacteremia, with 70% of the positive cultures isolated from patients with neutropenia (neutrophil count ≤ 1000 cell/µL). Our findings are consistent with studies in high and low resource settings demonstrating the risk of bacteremia in cancer patients who have febrile neutropenia [23, 28]. We also found that nearly three quarters of the positive bacteria isolates cultured were from patients with hematologic malignancies. Patients who have hematologic malignancies have been reported in other studies to be more likely to have a bacteremia compared to patients with solid cancers because of the immunosuppressive effects of the cancer as well as the intensive chemotherapy used to treat hematologic malignancies [28]. Although we did not find a statistically significant difference in risk of bacteremia between hematologic and solid tumor patients, perhaps due to the small sample size of our study, further evaluation of the specific risk of infection among hematologic patients in our setting is likely clinically important and warrants further study.

### Conclusion

This study showed that patients with neutropenia and underlying hematological malignancy are vulnerable to developing bacteremia, and multidrug resistant Gram-negative bacteria are the main cause of bacteremia in febrile cancer patients in Uganda. There is need for ongoing antimicrobial surveillance at cancer centers in SSA to guide antimicrobial therapy and support the development of infection control and antimicrobial stewardship programs at these institutions.

## Limitations

Our study

- Was limited by its cross-sectional design and relatively small sample size.
- Did not evaluate the risk factors and outcomes such as mortality associated with bacteremia and antimicrobial resistance including chemotherapy received, duration of neutropenia and antibiotic prophylaxis.
- Did not explore molecular mechanisms of resistance and relatedness among isolates.

## Data Availability

All data generated and analyzed during this study are included in this published article

## Abbreviations

SSA: sub-Saharan Africa
LMIC: low-middle-income countries
UCI: Uganda Cancer Institute
CLSI: Clinical & Laboratory Standards Institute
ESBL: extended spectrum beta-lactamase
